# Bilateral Avascular Necrosis of the Femoral Head in a Patient With Sickle Cell Trait: A Case Report

**DOI:** 10.7759/cureus.105265

**Published:** 2026-03-15

**Authors:** Saeed Nasher, Suhaip Alkamali, Assad Ali Alhadi

**Affiliations:** 1 Internal Medicine/Hematology, Taiz University Faculty of Medicine and Health Science, Taiz, YEM; 2 Department of Internal Medicine, Saeed Thabet Nasher Hospital, Taiz, YEM; 3 Radiology, Ibb Scan Center, Ibb, YEM

**Keywords:** avascular necrosis, case report, femoral head, hyperviscosity, osteonecrosis, sickle cell trait, vaso-occlusion, yemen

## Abstract

Sickle cell trait (SCT), historically regarded as a benign carrier state, may nonetheless be associated with severe complications such as bilateral avascular necrosis (AVN) of the femoral head in select individuals. This risk is often underrecognized, particularly in regions with high SCT prevalence, leading to delayed diagnosis and management. We report the case of a 43-year-old Yemeni man who presented with progressive bilateral hip pain over eight months without traditional risk factors except smoking. His family history was notable for sickle cell disease and trait. Physical examination revealed painful, restricted hip movement and mild splenomegaly. Laboratory testing confirmed SCT with an HbS level of 33.9% and a normal hemoglobin concentration of 16.5 g/dL. Initial radiographs were unremarkable, but MRI demonstrated bilateral femoral head AVN with marrow edema and subchondral changes. While alternative causes, including thrombophilia, were not fully excluded, conservative management was unsuccessful due to poor adherence, necessitating surgical referral. In conclusion, this case adds to emerging evidence that SCT may contribute to severe orthopedic morbidity in the presence of additional risk factors such as smoking. We advocate for a high index of suspicion for established causes of AVN and early MRI in symptomatic patients to prevent diagnostic delay and irreversible joint damage.

## Introduction

Avascular necrosis (AVN), also known as osteonecrosis, is a debilitating pathological condition resulting from the temporary or permanent interruption of the blood supply to bone tissue [1]. This ischemic insult leads to cessation of oxidative phosphorylation, subsequent cellular death, and eventual loss of trabecular bone structural integrity [1]. The femoral head is particularly vulnerable to AVN due to its unique "watershed" vascular supply, where impaired blood flow can result in subchondral collapse and secondary osteoarthritis [2]. The etiology of AVN is multifactorial, including trauma, chronic corticosteroid use, excessive alcohol consumption, systemic lupus erythematosus, thrombophilic and other hypercoagulable states, and hemoglobinopathies [3,4].

Within hemoglobinopathies, AVN is a hallmark complication of sickle cell disease (SCD). Its pathophysiology is driven by polymerization of sickle hemoglobin (HbS), which causes erythrocytes to lose deformability and adopt a rigid, sickled shape. These sickled erythrocytes adhere more readily to vascular endothelium, promoting vaso-occlusion, bone marrow infarction, and medullary fat necrosis [5]. By contrast, AVN is exceptionally rare in individuals with sickle cell trait (SCT), the heterozygous carrier state for the βS allele, who typically maintain HbS levels below the threshold required for widespread sickling under physiological conditions [6].

Although SCT is traditionally considered benign, emerging evidence highlights that under extreme metabolic stress, hypoxia, or dehydration, SCT carriers may develop severe complications such as splenic infarction and renal medullary carcinoma [7,8]. A "second hit" hypothesis has been proposed wherein additional stressors create conditions conducive to sickling in otherwise asymptomatic carriers [7,9]. AVN remains a rare but underreported complication in this population, with only isolated cases documented in the literature [4,10]. Herein, we report the unusual case of bilateral femoral head AVN in a patient with SCT and discuss the diagnostic challenges, the importance of excluding alternative etiologies, and the limitations in establishing causality from a single observation.

## Case presentation

A 43-year-old Yemeni male presented to our oncologic clinic with an eight-month history of progressive, bilateral hip pain. The pain was mechanical in nature, localized to the groin with radiation to the lower back and knees. Symptoms were significantly exacerbated by weight-bearing and only partially relieved by rest and over-the-counter analgesics.

The patient's past medical history was unremarkable except for chronic smoking (approximately 20 pack-years); he specifically denied previous sickle cell crises, trauma, chronic corticosteroid use, significant alcohol consumption, or travel to high-altitude regions. The family history was significant for hemoglobinopathies, including SCD in a first-degree cousin and SCT in a sibling. Occupationally, the patient had been a manual laborer for 15 years before transitioning to a sedentary administrative role eight years prior to presentation.

On physical examination, the patient was afebrile and hemodynamically stable. No pallor, jaundice, or lymphadenopathy was observed. Abdominal palpation revealed mild, non-tender splenomegaly. Orthopedic assessment of the hips demonstrated significant tenderness over both joint lines. Active and passive ranges of motion were markedly restricted and painful, particularly during internal and external rotation. Distal neurovascular status in the lower extremities was intact.

Initial laboratory investigations revealed a hemoglobin level of 16.5 g/dL, which is within the normal reference range for an adult male (13.5-17.5 g/dL). A peripheral blood smear showed no evidence of sickled cells. However, a positive solubility test (sickling test) prompted further analysis via high-performance liquid chromatography (HPLC). The HPLC confirmed a diagnosis of SCT with the following profile: HbS 33.9%, HbA 55.6%, HbA2 4.2%, and HbF 1.6% (Table 1). These findings effectively excluded SCD (HbSS) and other compound heterozygous states like S/β-thalassemia. It is important to note that a comprehensive evaluation for thrombophilia, coagulation disorders, or metabolic bone disease was not performed; this represents a significant limitation.

**Table 1:** Summary of laboratory investigations at presentation Abbreviations: HPLC: high-performance liquid chromatography; SCT: sickle cell trait

Test	Patient's value	Reference range
Complete blood count		
Hemoglobin (Hb)	16.5 g/dL	13.5–17.5 g/dL
Mean corpuscular volume (MCV)	82.3 fL	82–98 fL
White blood cell count (WBC)	3.6 × 10³/µL	4.0–11.0 × 10³/µL
Platelet count	205 × 10³/µL	150–450 × 10³/µL
Reticulocyte count	1.2%	0.5–2.0%
Inflammatory markers		
Erythrocyte sedimentation rate (ESR)	12 mm/hr	0–15 mm/hr
C-reactive protein (CRP)	< 6 mg/dL	<6 mg/dL
Biochemistry		
Alanine aminotransferase (ALT/SGPT)	37 U/L	≤31 U/L
Serum calcium	8.4 mg/dL	8.9–10.7 mg/dL
Specialized hematological tests		
Sickling test	Positive	Negative
Hemoglobin electrophoresis (HPLC)		
Hemoglobin S (HbS)	33.9%	0%
Hemoglobin A (HbA)	55.6%	95–98%
Hemoglobin A2 (HbA2)	4.2%	1.5–3.5%
Hemoglobin F (HbF)	1.6%	<2.0%
Peripheral Blood Smear	No sickled cells, schistocytes, or spherocytes noted	

**Figure 1:**
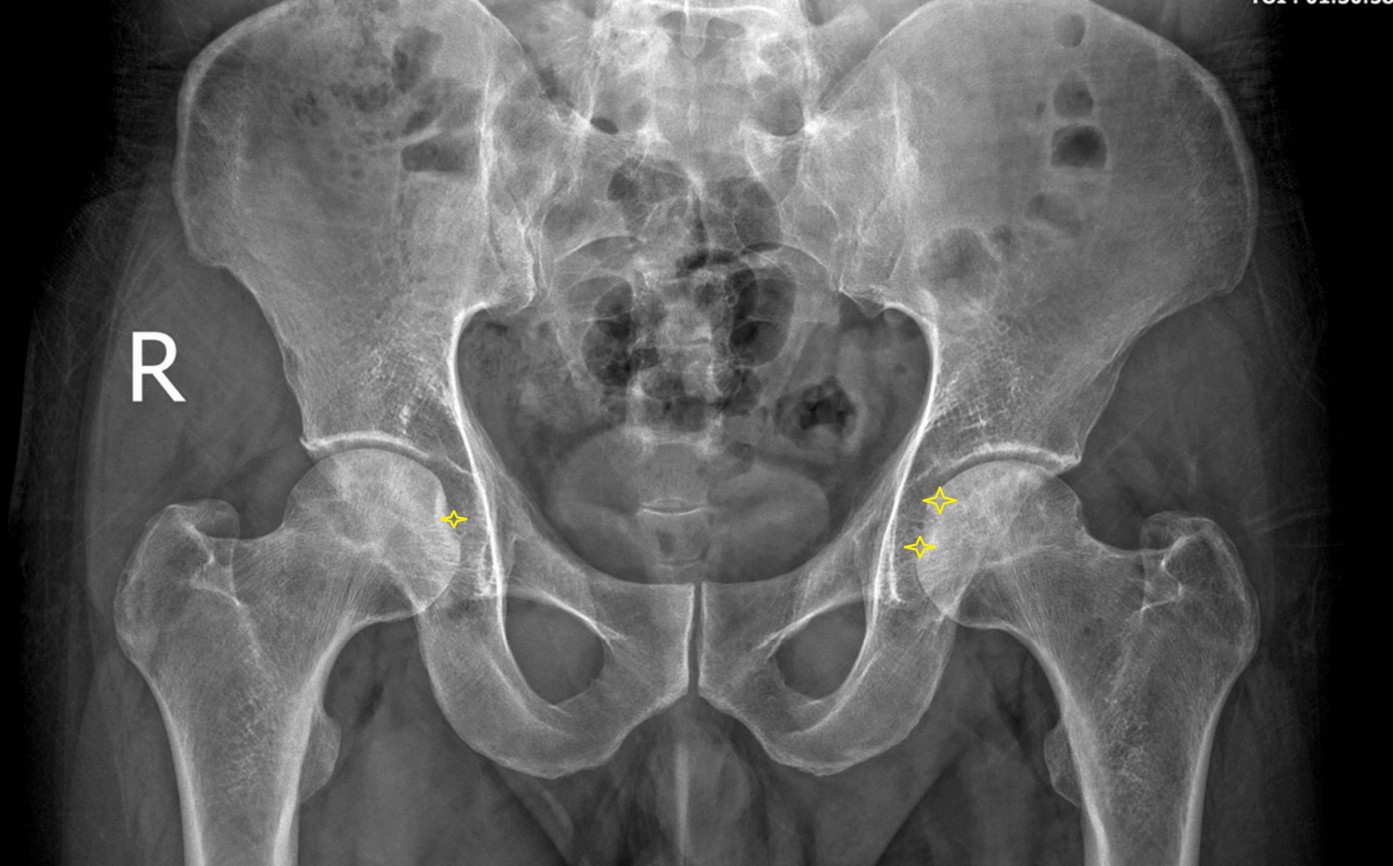
Anteroposterior (AP) pelvic radiograph of a 43-year-old male with sickle cell trait. A follow-up at three months revealed a faint, curvilinear radiolucent line parallel to the articular surface of the left femoral head, known as the "crescent sign" (asterisk). This finding indicates subchondral fracture and impending articular collapse, consistent with Ficat and Arlet stage III.

Initial anteroposterior (AP) radiographs of the pelvis appeared unremarkable. However, follow-up radiographs three months later revealed subtle bilateral changes, including increased subchondral sclerosis and a faint fissure line (crescent sign) in the left femoral head, indicating early structural compromise (Figure 1).

**Figure 2:**
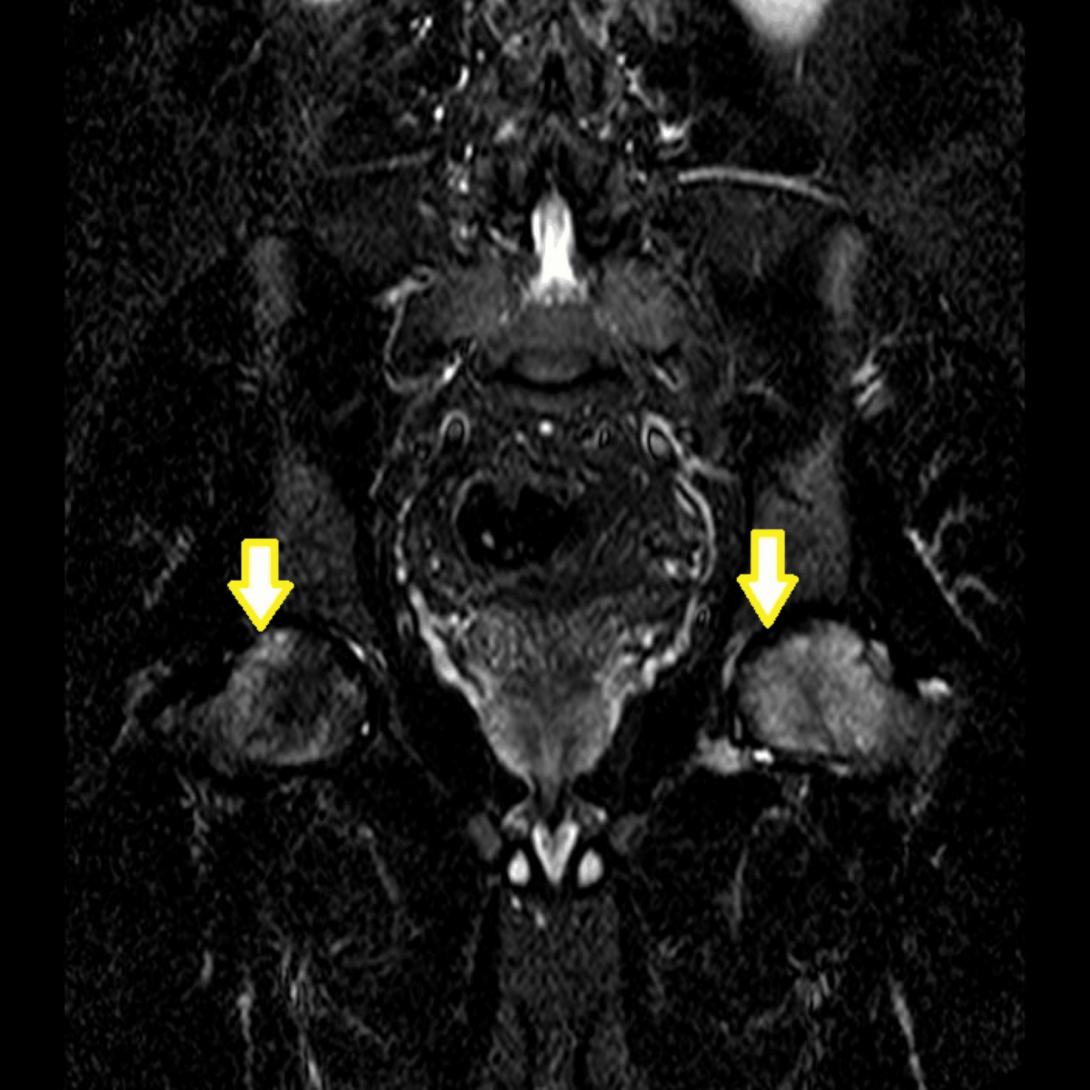
Coronal T2-weighted short tau inversion recovery (STIR) MRI of the pelvis. The image reveals extensive, high-signal intensity bone marrow edema involving both femoral heads. Note the presence of the pathognomonic "double-line sign"; a low-signal peripheral rim of sclerosis with an inner high-signal band of granulation tissue, demarcating the necrotic segment. These findings confirm bilateral avascular necrosis with early cystic changes and volume loss, more pronounced on the left side.

Magnetic resonance imaging (MRI) was performed to provide a definitive assessment. T1-weighted and T2-weighted STIR sequences demonstrated bilateral AVN characterized by extensive bone marrow edema and subchondral cystic formations. The images revealed a pathognomonic "double-line sign" on T2 sequences, consistent with Ficat and Arlet stage III on the left and stage II on the right (Figure 2). Abdominal ultrasound subsequently confirmed the presence of splenomegaly.

The final diagnosis was bilateral AVN of the femoral heads. The patient was initially managed conservatively with monthly intravenous bisphosphonates (zoledronic acid), optimized analgesia, and strict instructions for non-weight-bearing.

At the three-month follow-up, the patient reported only marginal symptomatic relief. He admitted to poor adherence to non-weight-bearing restrictions due to the socioeconomic demands of his occupation. Repeat imaging demonstrated progression of the necrotic lesions and early collapse of the articular surface. Given the failure of conservative therapy and the risk of rapid joint destruction, the patient was referred for advanced orthopedic consultation to discuss surgical options, including core decompression or total hip arthroplasty (THA). Unfortunately, the patient was lost to follow-up after this referral; despite multiple attempts to contact him and his family, no further clinical or imaging data were obtainable.

## Discussion

AVN is a progressive condition caused by reduced blood flow to bone tissue, leading to structural collapse and joint dysfunction [2]. It most often impacts the femoral head because it relies on terminal blood flow and has limited alternative circulation [1]. In patients with SCD, AVN commonly arises from repeated vaso-occlusive episodes that restrict blood flow and diminish oxygen supply to the bone [5].

This case involves a patient who discovered SCT incidental to a diagnosis of bilateral AVN. Although AVN is a known complication of SCD, it remains exceptionally rare in individuals with the SCT genotype, with only isolated cases reported in recent literature [4,10,11]. Spontaneous vaso-occlusive events are exceptionally rare in SCT under normal physiology, typically requiring co-factors or extreme stressors such as high-altitude exposure or severe dehydration [12]. Unless SCT is inherited alongside another hemoglobinopathy or a condition that promotes blood clotting, vaso-occlusive events are uncommon [6,12]. Therefore, it is important to investigate known risk factors such as alcohol abuse, glucocorticoid therapy, or trauma before attributing AVN to SCT [3,4].

In this patient, chronic smoking (approximately 20 pack-years) was the only identifiable risk factor. Smoking can cause endothelial dysfunction and chronic hypoxia, which may have synergized with SCT as a genetic modifier [13]. Although ultrasound revealed splenomegaly, this finding should be approached with caution rather than viewed as a sign of active disease. SCT is typically asymptomatic with normal splenic function; major splenic issues are rarely noted outside specific situations like high-altitude exposure, extreme physical exertion, or conditions such as hereditary spherocytosis [14]. The presence of both AVN and splenomegaly in this case likely reflects concurrent ischemic processes rather than a direct cause-and-effect relationship, potentially supporting a "second hit" model wherein additional stressors contribute to microvascular compromise in susceptible carriers [6,7].

The development of splenic and bone issues in SCT relates to low oxygen levels in localized areas, which are seldom reached under normal conditions [14]. Hemoglobin S (HbS) polymerization in heterozygotes usually requires severe physiologic stressors, such as extreme dehydration or systemic acidosis, to transform the silent carrier state into a vaso-occlusive syndrome [6]. SCT may have increased vulnerability to restricted blood flow in the femoral head, worsened by smoking, but clear triggers for bilateral necrosis remain elusive. This situation highlights the need for genetic testing in unusual SCT cases to identify potential modifiers that might trigger vaso-occlusion in otherwise healthy carriers [7].

For SCT carriers with bilateral AVN, excluding alternative etiologies through targeted testing is prudent prior to considering a contributory role for the hemoglobinopathy. Routine screening for thrombophilia, coagulation disorders, or metabolic bone disease is not universally mandated, as AVN remains exceptionally rare in the heterozygous HbAS genotype [9]. However, its presence warrants a risk-stratified investigation into more common drivers of osteonecrosis [3,4]. Additional testing should be individualized based on clinical suspicion and guided by factors such as family history of thrombosis, recurrent pregnancy loss, autoimmune markers, or atypical presentation.

This case involved a notable family history of hemoglobinopathies, yet the absence of targeted testing for co-inherited modifiers or alternative AVN etiologies represents a key limitation. For future cases, we recommend thorough evaluation for established causes of AVN, with SCT considered a diagnosis of exclusion only after primary etiologies are ruled out [9]. This approach prioritizes the identification of actionable comorbidities and prevents oversight of common osteonecrosis etiologies due to the incidental discovery of a heterozygous carrier state.

Recent case reports confirm AVN can occur in SCT when additional risk factors are present. Keeling et al. described bilateral femoral head AVN in a patient with SCT and unexplained erythrocytosis [15]. Sanders reported a case requiring bilateral hip arthroplasty [11]. Brown et al. described three SCT patients with multifocal osteonecrosis, all having additional risk factors [13]. Al-Zaher et al. documented bilateral AVN in a patient with SCT and chronic alcohol use, supporting SCT's role as a modifier rather than the sole cause [10]. These findings align with this case, where chronic smoking likely played a primary role, with SCT potentially increasing risk through microvascular effects. This emphasizes the importance of holistic evaluation of SCT patients, with close attention to modifiable risk factors when they present with unexplained joint pain.

Management of AVN varies based on disease severity and stage. Initially, treatment focuses on non-surgical options, including pain relief, anti-inflammatory medications, physical therapy, and activity modification [16]. In early stages with intact femoral head morphology, core decompression may relieve intraosseous pressure and promote revascularization. Joint-preserving procedures include bone grafting, implants, and osteotomies [16]. In advanced collapse, THA becomes appropriate, offering significant pain relief and functional improvement despite elevated infection risk reported in hemoglobinopathy populations [11,13]. Given this patient's surgical hesitancy and subsequent loss to follow-up, conservative management with monitoring remains suitable, with THA reevaluation if progression occurs.

This case adds to the sparse literature on AVN in SCT and underscores the need for careful evaluation of joint pain, particularly when modifiable risks are present. Early detection, risk factor modification (notably smoking cessation), and personalized management can preserve function and avert disability. Further research with larger cohorts is needed to clarify potential associations between SCT and AVN and to optimize evidence-based management strategies for this rare presentation.

## Conclusions

This case illustrates bilateral AVN in a patient with SCT without full SCD or typical vaso-occlusive crises, highlighting SCT's potential as a disease modifier rather than primary driver. Chronic smoking likely accelerated progression via endothelial dysfunction and chronic hypoxia. The finding of mild splenomegaly may reflect subclinical microvascular trapping, consistent with the "second hit" model proposed for other SCT-related complications. However, the absence of a comprehensive evaluation for alternative etiologies represents a significant limitation, and SCT should remain a diagnosis of exclusion. Early imaging, risk factor cessation, and personalized management optimize outcomes while preparing for potential surgical intervention. These findings reinforce clinical vigilance for ischemic complications in SCT carriers with co-existing risk factors, challenging assumptions of complete benignity without overattributing causation.
